# Current practice in analysing and reporting binary outcome data—a review of randomised controlled trial reports

**DOI:** 10.1186/s12916-020-01598-7

**Published:** 2020-06-08

**Authors:** Ines Rombach, Ruth Knight, Nicholas Peckham, Jamie R. Stokes, Jonathan A. Cook

**Affiliations:** 1 Nuffield Department of Orthopaedics, Rheumatology and Musculoskeletal Sciences (NDORMS), University of Oxford, Oxford, UK; 2 Oxford Clinical Trials Research Unit, NDORMS, University of Oxford, Oxford, UK; 3 Centre for Statistics in Medicine, NDORMS, University of Oxford, Oxford, UK

**Keywords:** Clinical trials, Binary outcomes, Incomplete data, Statistical methods, Reporting guidance

## Abstract

**Background:**

Randomised controlled trials (RCTs) need to be reported so that their results can be unambiguously and robustly interpreted. Binary outcomes yield unique challenges, as different analytical approaches may produce relative, absolute, or no treatment effects, and results may be particularly sensitive to the assumptions made about missing data. This review of recently published RCTs aimed to identify the methods used to analyse binary primary outcomes, how missing data were handled, and how the results were reported.

**Methods:**

Systematic review of reports of RCTs published in January 2019 that included a binary primary outcome measure. We identified potentially eligible English language papers on PubMed, without restricting by journal or medical research area. Papers reporting the results from individually randomised, parallel-group RCTs were included.

**Results:**

Two hundred reports of RCTs were included in this review. We found that 64% of the 200 reports used a chi-squared-style test as their primary analytical method. Fifty-five per cent (95% confidence interval 48% to 62%) reported at least one treatment effect measure, and 38% presented only a *p* value without any treatment effect measure. Missing data were not always adequately described and were most commonly handled using available case analysis (69%) in the 140 studies that reported missing data. Imputation and best/worst-case scenarios were used in 21% of studies. Twelve per cent of articles reported an appropriate sensitivity analysis for missing data.

**Conclusions:**

The statistical analysis and reporting of treatment effects in reports of randomised trials with a binary primary endpoint requires substantial improvement. Only around half of the studied reports presented a treatment effect measure, hindering the understanding and dissemination of the findings. We also found that published trials often did not clearly describe missing data or sensitivity analyses for these missing data. Practice for secondary endpoints or observational studies may differ.

## Background

Randomised controlled trials (RCTs) are commonly conducted to provide an evidence base for current and new treatments, inform evidence-based healthcare, and improve patients’ outcomes and welfare. Binary outcomes are those that can take only one of two values, such as treatment failure or success, or mortality (dead or alive). Many trials have a binary outcome as one of the key measures used to compare treatments. Charles et al. [1] found that around half of trials calculated their sample size based on a binary outcome. Trials using binary outcomes have different statistical and other considerations to trials using other outcome types, such as continuous and time-to-event.

As for all outcomes, the analysis and reporting of the findings of binary outcomes is clearly a key aspect of good scientific practice and is critical for maximising the value of the research. However, to our knowledge, little research has been carried out on the analysis of binary endpoints in clinical trials. This is surprising, as their use has implications for the analysis planned and the reporting. Numerous statistical analysis approaches exist for analysing binary outcomes, such as logistic regression and, more recently, Poisson regression with appropriate calculation of standard errors. Anecdotally, statistical analysis methods that do not produce an estimate of the effect size and only produce a *p* value (e.g. chi-squared-style test) seem to be more commonly used. Related to this, the target difference used in the sample size calculation can be based on a relative (e.g. risk ratio of 0.75) or absolute (e.g. reduction from 80 to 60%) difference in the treatment effect of the binary outcome. Statistical adjustment for a covariate when analysing binary outcomes (and also time-to-event outcomes) should be considered carefully as unadjusted and adjusted analyses estimate different treatment effects [2–4].

The Consolidated Standards of Reporting Trials (CONSORT) statement [5] recommends reporting both the relative treatment effect (e.g. odds ratio or relative risk) and an absolute treatment effect (e.g. risk difference) for binary outcomes. Presenting both effects arguably gives a more complete picture of the results and their implications than reporting just one. For example, the doubling of an event rate (i.e. relative risk) will have more relevance for public health if the outcome has a high overall risk than if the outcome has a low overall event rate. For a given relative treatment effect, the absolute risk difference would also be much larger if the event had a high overall event rate, compared with a low overall event rate. Nevertheless, most statistical analysis methods that do directly produce an effect size estimate provide only one estimate, usually a relative difference.

Missing data are also handled differently for binary and other outcomes. Missing data in clinical trials are commonly analysed using available cases. Imputation methods, including simple imputation, multiple imputation, and worst-case scenarios, are used less frequently in primary analyses [6–8]. All approaches for missing data make strong, untestable assumptions about the underlying missing data mechanism. It is unclear if and how such analyses are done. Sensitivity analyses to assess the impact of these assumptions are recommended, but rarely used [6–8].

How binary outcomes are analysed in clinical trials and the findings reported is therefore of much interest. In this paper, we report a systematic review of the statistical analysis of binary outcomes in recently published RCTs. We focus on the methods used to analyse binary primary outcomes, how missing data are handled, and how the findings are reported.

## Methods

The literature was searched for reports of RCTs published in January 2019 (e-publication or print). Studies were eligible if they reported the findings of randomised controlled trials with a binary primary outcome. We did not limit the inclusion criteria to any particular condition, intervention or patient group. We excluded papers classed as meta-analyses, comments, letters, editorials, or news; animal experiments; and studies not in humans. We did not restrict the search by journal or disease area. Only articles written in English were considered.

We developed a search strategy for PubMed (see Additional file 1: ‘Summary of search strategy and papers identified’ for full details of the search terms used). The search was performed on 18 April 2019 by the lead author (IR), to allow sufficient time for indexing.

The target sample size was 200 papers, as this was considered a sufficiently large number of papers to produce a generalisable assessment of current practice, in line with similar studies. This target was also considered sufficient to let us measure binary outcomes to a confidence interval (CI) width of 0.08 to 0.14, depending on the event rate, based on Wilson’s CI method [9].

Titles and abstracts were screened by the lead author (IR) to identify reports of two-arm, parallel-group, individually randomised trials with a binary primary endpoint. The primary endpoint had to be clearly described as such in the paper, used to determine the sample size calculation, or was referred to in the aims and objectives of the paper. We excluded cluster, cross-over, and multi-arm (> 2 arms) trials and papers reporting a principal analysis based on time-to-event data. We also excluded pilot and feasibility studies and papers that were not primary reports of clinical trials.

Titles and abstracts were screened in chronological order (by date of publication listed in EndNote) until 200 eligible papers had been identified.

We used a standardised data extraction form to collect information (see Additional file 2 for a list of items) on the characteristics of the included studies, the analysis, and reporting approaches.

All authors contributed to the development of the data extraction forms (using Microsoft Excel) and piloted them on a number of papers. All authors contributed to the data extraction, and each reviewer confirmed that their allocated papers matched the inclusion criteria, based on a full-text review. To assess the reliability of the extraction, duplicate extraction was performed for 10% of the sample. Discrepancies were resolved by the lead author. Single data extraction was performed for the remaining studies.

Information on study characteristics (including disease area, journal, trial design, single vs. multicentre, sample size, and funding), principal analysis methods, additional analyses of the primary endpoint, amounts of missing data, and participants included in the analysis was extracted from the final set of papers. We assessed whether relative and absolute risks and their CIs were presented and whether *p* values were given. We also extracted the method of handling missing data in the principal analysis and whether relevant sensitivity analyses were performed. Full details of all items extracted can be found in the supplementary material.

We generated descriptive statistics using frequency and percentage for categorical data and median, interquartile range, mean, and standard deviation for continuous data. Frequencies and percentages were also generated. We also collected data on how many studies provided *p* values for the comparison between groups. 95% confidence intervals (CI) using Wilson's methods were calculated for the key binary measures of interest. The association between journal impact factors and whether or not at least one treatment effect was reported were based on a logistic regression model; an odds ratio with 95% CI was calculated. Impact factor was used as the single continuous covariate in this model.

Analyses and summaries were generated in Stata/IC version 15.

## Results

**Fig. 1:**
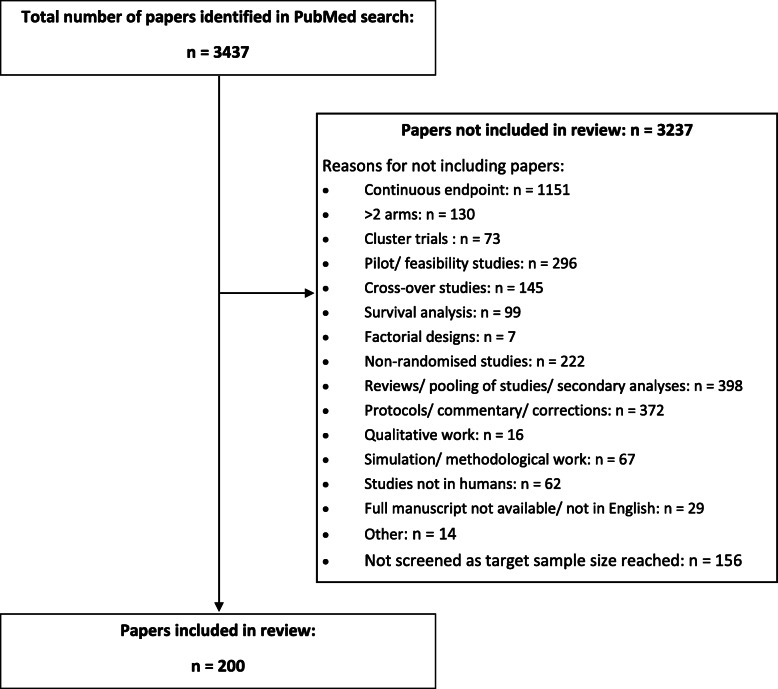
PRISMA flow chart for the literature search

Figure 1 shows the number of articles obtained from the literature search, articles excluded and why, and articles included in the study. Two hundred articles, covering a wide range of disease areas and journals, were included in this review.

The most commonly included disease areas were obstetrics and gynaecology (*n* = 32, 16%), gastroenterology, oncology, and infectious diseases (each *n* = 24, 12%). Other medical specialities, including cardiology, critical care, dermatology, paediatrics, public health and policy interventions were also covered by the studies. The studies were published in journals with impact factors ranging from 0.4 to 70.7, with a mean of 8.7 (standard deviation 15.8). The median was 3.4, with an interquartile range from 2.2 to 6.0.

The double data extraction for 10% of the included papers demonstrated good agreement between the reviewers. Seventeen key items were extracted for each of the 20 papers. Less than 10% discrepancies were found (31/340), and these tended to be very minor.

Additional characteristics of the studies included in this review, i.e. trial design, sample size, whether they included multiple centres, and the main funding source, are shown in Table 1.

**Table 1:** Characteristics of the 200 studies included in this review

Trial design	Superiority	157 (79%)
	Non-inferiority	33 (17%)
	Equivalence	3 (2%)
	Unclear	7 (4%)
Size	Median = 145, interquartile range = 82–400	
Multicentre	Yes	110 (55%)
	No	83 (42%)
	Unclear	7 (4%)
Funding	Public, charity, or public and charity	68 (34%)
	Solely industry	38 (19%)
	Others (include combinations)	40 (20%)
	No stated funding	54 (27%)

Two of the 200 studies reported that no analyses of the primary endpoint were performed.  Of the 198 studies that reported an analysis, almost two thirds (*n* = 127, 64%) reported using chi-squared-style tests as their principal analysis method. Logistic regression was reported in only 22 (11%) papers. The principal analysis method was not clear in 24 (12%) papers based on the main text. Ninety per cent of the abstracts did not indicate the analytical methods used for the primary endpoint. Details of the analyses reported are provided in Table 2. Adjustment of the principal analysis was reported in 32 (18%) of the 174 studies that had specified the analytical method.

**Table 2:** Principal analysis method reported for the primary binary outcome used in the 200 included studies

	Reported in the main text (*n* = 200)	Reported in the abstract (*n* = 200)
Chi-squared-style tests^1^	127 (64%)	13 (7%)
Logistic regression	22 (11%)	3 (2%)
Looking at confidence interval limits^2^	8 (4%)	3 (2%)
Binomial regression	7 (4%)	1 (1%)
Others^3^	10 (5%)	0 (0%)
Not reported	24 (12%)	180 (90%)
No analysis^4^	2 (1%)	0 (0%)

^1^Including Fisher’s exact and Mantel-Haenszel test

^2^‘Looking at confidence intervals’ refers to where the assessment of non-inferiority was made by comparing the upper or lower limits of the confidence interval, as appropriate, to the non-interiority margin

^3^These include Poisson models, exact binomial test, tests for non-inferiority (including Farrington-Manning), and Newcombe’s method

^4^One study reported no events and therefore did not perform the planned principal analysis. One study described a composite primary endpoint, which was not reported in the paper; the components of the composite endpoint were reported separately

In total, 41 papers (21%, Wilson’s confidence interval (CI) 15 to 27%) used a primary analytical method that was able to produce a treatment effect estimate.

Seventeen per cent of studies reported both an adjusted and unadjusted analysis for the primary binary endpoint.

Table 3 shows the number of studies that reported absolute effects, relative effects, or both, referring to all analyses of the primary binary endpoint, and not just the principal analysis. One hundred and nine studies of the 198 that reported a statistical analysis (55%, Wilson’s 95% CI 48 to 62%) provided at least one treatment effect estimate. A *p* value for a test of a difference between the groups was given in 164 (83%, Wilson’s 95% CI 77 to 87%) studies. Twenty studies provided treatment effects without *p* values. A higher journal impact factor was statistically significantly associated with at least one treatment effect estimate (either relative or absolute) being reported, odds ratio = 1.15 (95% CI 1.05 to 1.28).

**Table 3:** Reporting of treatment effects, confidence intervals, and *p* values in the 198 studies that performed a statistical analysis

	Full text (*n* = 198)	Abstract (*n* = 198)
Reporting of statistical analysis		
Any treatment effect measure^1^	109 (55%)	75 (38%)
No treatment effect measure, *p* value only	75 (38%)	86 (43%)
No statistical analysis result reported	14 (7%)	37 (19%)
Reporting of treatment effects^2^		
Relative treatment effect only (point estimate)	55 (28%)	42 (21%)
Point estimate and CI	55 (28%)	42 (21%)
Absolute treatment effect only (point estimate)	33 (17%)	26 (13%)
Point estimate and CI	30 (15%)	25 (13%)
Both relative and absolute treatment effects reported (point estimate)^3^	18 (9%)	7 (4%)
Point estimates and CIs	16 (8%)	6 (3%)

Numbers refer to any analyses for the primary binary outcome in the report and are not limited to the only principal analysis

*CI* confidence interval

^1^Including papers that reported an estimate of an absolute or relative effect measure (point estimate and/or confidence interval)

^2^Three studies reported confidence intervals, but no point estimate (full text only). These studies were counted in ‘reporting of statistical analysis’, but not in ‘reporting of treatment effects—relative/absolute treatment effects only (point estimate)’ and ‘both relative and absolute treatment effects reported (point estimate)’

^3^Where both absolute and relative treatment effect estimates were presented, different statistical methods were used to obtain these estimates; no papers described that transformations to obtain an absolute effect from a relative one, or vice versa, were used

Some primary follow-up data were missing in at least 140 (70%, Wilson’s 95% CI 63 to 76%) studies, although the extent of missing data was only clear in 118 (59%) studies. The studies that had missing primary outcome data and were clear about how much missing data there was had a median missing data rate of 5% (interquartile range 2 to 14%).

Table 4 shows the approaches used to handle missing data in the primary analyses and whether an appropriate sensitivity analysis was performed for missing data, if any existed. Of the 140 studies that reported missing data, 96 (69%) reported using an available case analysis; imputation and best/worst case scenarios were reported in 30 studies (21%).

**Table 4:** Handling of missing data in the principal analysis of the 140 studies that reported some missing data in their primary outcome and performed an analysis

Approach to handling missing data in the principal analysis	
Available cases	96 (69%)
Multiple imputation	9 (6%)
Worst-case/best-case scenario	18 (13%)
Last observation carried forward	2 (1%)
Other^1^	1 (1%)
Unclear	14 (10%)
Performance of appropriate^2^ sensitivity analysis for missing data	17 (12%)

^1^In one study, missing outcomes were imputed by independent assessors using a pre-defined set of rules provided in a supplementary appendix

^2^Defined as an analysis that varies the assumptions made about the underlying missing data mechanism

Seventeen of the relevant papers where missing data were reported (12%, Wilson’s 95% CI 8 to 19%) performed an appropriate sensitivity analysis for missing data.

## Discussion

Our review showed that the statistical analysis and reporting of treatment effects in reports of randomised trials with a binary primary endpoint requires substantial improvement. Most of the trials used a principal analysis method that did not provide an estimate of the treatment effect. Less than half of the reviewed studies reported an estimate of the treatment effect from any analysis of the primary endpoint (principal or secondary). Disappointingly, 12% did not clearly state which principal analysis method was used. Only 8% reported both a relative and absolute treatment effect with corresponding CIs as recommended by the CONSORT 2010 statement [5]. This low adherence to the CONSORT reporting guideline is perhaps disappointing, considering that many biomedical journals endorse the CONSORT guideline. However, it probably reflects, at least partially, the complexity of implementing the guideline.

Possible solutions exist to remedy these shortcomings in conduct and reporting. The most commonly used principal analysis method was a chi-squared-style test. The use of these tests should be discouraged in favour of alternative statistical analysis methods such as logistic or binomial regressions, which produce estimates of the effect size. This may reflect a limitation in medical statistics training to health professionals, which can overemphasise statistical testing to the detriment of a more rounded approach to statistical inference. It may also reflect a more profound problem in modern research: innovation is more likely to be rewarded than evaluation and adoption of better practice. Strangely, although quantification of the treatment effect and uncertainty is the overwhelming practice in trial meta-analyses [10], it is often not done in trials themselves.

To meet the concerns raised here, we recommend using a statistical analysis method that estimates the magnitude of the treatment effect and quantifies the uncertainty (e.g. CI) to analyse the primary binary outcome of a randomised trial, whether as the principal method or a pre-specified secondary analysis. This is eminently achievable. Even for smaller trials with fewer events, the magnitude of the treatment effect can be readily quantified and uncertainty with CIs calculated as long as there is at least one event [11, 12]. Reporting of the corresponding *p* value also has its place, at least for the principal analysis of the primary outcome [13].

Methods that allow quantification of the treatment effect and related uncertainty are readily available, such as the calculation for the unadjusted odds ratio and its CI [14] and logistic regression for the adjusted odds ratio.

Reporting the magnitude of the treatment effect is vital for communicating the trial findings in a meaningful, transparent way to all stakeholders, particularly patients and members of the public. Just identifying a statistically significant difference is not enough to confirm action [15], and finding the absence of evidence of a statistical difference is not enough to conclude no difference between treatments. The crude dichotomisation of findings has thus been heavily criticised, and alternative approaches and remedies suggested [16]. Perhaps, the most successful initiative in this area has been driven by journals like the *BMJ* and others, who early on promoted reporting the uncertainty around estimates (typically with a CI) [12]. However, trial reports often still do not state an estimate of the treatment effect and/or quantify the uncertainty around the estimate. Presumably, many researchers feel the reader can calculate the uncertainty themselves by looking at the observed event rate in each group. However, this stance is not acceptable in our view, given the importance of this value as the main study finding.

Different audiences may be more used to interpreting relative or absolute risk, although effects tend to be overestimated when presented as relative risks [5]. The practicalities of calculating both relative and absolute treatment effects are surprisingly complex. The statistical methods typically used directly calculate one or the other. Some researchers may then believe that they need to use different statistical methods to be able to report both relative and absolute treatment effects, which was the approach chosen in all studies that presented both relative and absolute treatment effect estimates. Performing more analyses raises multiplicity issues: the chance of obtaining spurious significant results increases with the number of tests performed, as does the potential for selecting the more favourable results if the principal analysis is not pre-specified. Recent work on sample size calculations [17, 18] has highlighted the need to clarify what the target difference is when designing the trial.

Only 8% of the included studies reported both an absolute and relative treatment effect with corresponding CIs, as recommended by the CONSORT guidelines [5]. Similarly, only 10% reported the statistical analysis method used to analyse the primary outcome in the abstract. If the CONSORT guidelines are rarely followed in these regards, either adherence should be more strongly encouraged by journals and peer reviewers, or they should be relaxed. Although it may be helpful to present both relative and absolute treatment effects, providing estimates of the event rates by treatment arm with either type of treatment effect estimate can arguably also convey the full picture of the intervention without requiring two formal tests. Most statistical analysis methods implicitly or explicitly assess a relative treatment effect. They tend to be more precise in our experience, at least in terms of detecting a statistically significant difference, than methods that assess absolute effects. When interpreting relative treatment effects, researchers should bear in mind that odds ratios tend to be higher than risk ratios (in some cases substantially so), and thus, for any relative effect bearing in mind the anticipated control risk rate is critical [19–21].

The extent of missing data was generally insufficiently described, leading to a lack of clarity regarding how much outcome data were missing, and hence how reliable the results were. When data were missing, appropriate sensitivity analyses based on varying assumptions about the missing data mechanism, including worst-case scenarios, were rarely performed. Sensitivity analyses for missing data are particularly important for binary outcomes as even small changes in the numbers of events by treatment arm can change the treatment estimates or even the trial conclusions.

The large number of recently published clinical trial reports in a range of journals and clinical areas reviewed is a strength of the methodology used. The papers included are therefore representative of current practice and show that there are problems in published reports across journals and irrespective of markers of journal prestige (e.g. higher impact factors). While there is some indication that treatment effects are more likely to be reported in higher-impact journals, there was room for improvement in terms of reporting of statistical methods, treatment effect estimates, and missing data across the board. Our study was not designed in order to be able to look at the difference between particular subgroups, such as disease area or source of funding. Further research should investigate if there are differences in the analysis and reporting of binary outcomes by study characteristics so that focus and any strategies for improving reporting can be directed where they may be needed most.

There are a number of limitations to this work, which we aimed to mitigate where possible. We performed our database search in April 2019 for papers published in January 2019. It is possible that not all relevant papers were indexed by then, causing us to unintentionally exclude some papers from the review. However, due to our fairly large sample and otherwise broad inclusion criteria, we do not anticipate that this limitation will have biased our findings. As the target sample size was set at 200 papers, we did not include all of the papers identified by the literature search in the final review. However, we screened papers in their publication date order, as collected in EndNote, which we anticipate will have prevented bias. For most of the papers, data were extracted by only one reviewer. We performed double extraction by two independent reviewers for around 10% of the papers, which showed good agreement on the key items of treatment effects reported, statistical tests performed, and missing data. Although the sample size was substantial, the 200 studies may not reflect the full range of practices, particularly for less-researched areas. Previous reviews have demonstrated that the choice of outcome and practice can vary substantially by clinical area [1, 22, 23].

Our review was limited to English language publications. Although this is likely to be representative of most RCTs, practice for RCTs reported only in other languages may differ. We included only reports with primary binary outcomes. Although we have not investigated practice for secondary binary outcomes, we assume that the methods used for secondary outcomes will rarely be superior to those used for primary outcomes. However, we believe that the included studies do reflect current practice analysing binary outcomes in general.

More generally, this review did not consider other outcome types, such as time-to-event, ordinal, and continuous outcomes. In our view, similar reviews of these other outcomes would also be highly beneficial. We also restricted the review to randomised trials with a two-arm parallel-group design. Current practice for other RCT designs and observational studies evaluating treatments may differ.

## Conclusions

This study identified substantial room for improvement in the analysis and reporting of binary primary outcomes in RCTs. Binary endpoints were often analysed with a method that did not provide an estimate of the treatment effect or its related uncertainty inhibiting interpretation of findings. Published trials often did not clearly describe missing data or perform sensitivity analyses for these missing data. The main limitation of this research was its focus on binary primary outcomes in RCTs, excluding secondary outcomes. Our findings suggest that the analysis and reporting of binary endpoints in RCTs needs to improve.

## Supplementary information

**Additional file 1.** Summary of search strategy and papers identified.

**Additional file 2.** List of items extracted.

## Data Availability

Details of the search strategy and data extracted from the articles are included in the additional files.

## Abbreviations

CI: Confidence interval
CONSORT: Consolidated Standards of Reporting Trials
RCT: Randomised controlled trial

## References

[1] Charles P, Giraudeau B, Dechartres A, Baron G, Ravaud P (2009). Reporting of sample size calculation in randomised controlled trials: review. BMJ..

[2] Ford I, Norrie J (2002). The role of covariates in estimating treatment effects and risk in long-term clinical trials. Stat Med.

[3] Hernandez AV, Steyerberg EW, Habbema JD (2004). Covariate adjustment in randomized controlled trials with dichotomous outcomes increases statistical power and reduces sample size requirements. J Clin Epidemiol.

[4] Steyerberg EW, Bossuyt PM, Lee KL (2000). Clinical trials in acute myocardial infarction: should we adjust for baseline characteristics?. Am Heart J.

[5] Schulz KF, Altman DG, Moher D, Group C (2010). CONSORT 2010 statement: updated guidelines for reporting parallel group randomised trials. BMJ..

[6] Bell ML, Fiero M, Horton NJ, Hsu CH (2014). Handling missing data in RCTs; a review of the top medical journals. BMC Med Res Methodol.

[7] Rombach I, Rivero-Arias O, Gray AM, Jenkinson C, Burke O (2016). The current practice of handling and reporting missing outcome data in eight widely used PROMs in RCT publications: a review of the current literature. Qual Life Res.

[8] Wood AM, White IR, Thompson SG (2004). Are missing outcome data adequately handled? A review of published randomized controlled trials in major medical journals. Clin Trials.

[9] Piegorsch WW (2004). Sample sizes for improved binomial confidence intervals. Comput Stat Data Anal.

[10] Higgins JPT, Green S (editors). Cochrane Handbook for Systematic Reviews of Interventions Version 5.1.0 [updated March 2011]. The Cochrane Collaboration. 2011. Available from https://handbook.cochrane.org.

[11] Cook JA, Bunce C, Dore CJ, Freemantle N, Ophthalmic Statistics G (2015). Ophthalmic statistics note 6: effect sizes matter. Br J Ophthalmol.

[12] Gardner MJ, Altman DG (1986). Confidence intervals rather than P values: estimation rather than hypothesis testing. Br Med J (Clin Res Ed).

[13] Cook JA, Fergusson DA, Ford I, Gonen M, Kimmelman J, Korn EL, Begg CB (2019). There is still a place for significance testing in clinical trials. Clin Trials..

[14] Altman DG, Machin DB, Trevor N, Gardner MJ. (eds.). Statistics with confidence: confidence intervals and statistical guidelines, 2nd ed. London: BMJ Books; 2000.

[15] Wells G, Beaton D, Shea B, Boers M, Simon L, Strand V, Brooks P, Tugwell P (2001). Minimal clinically important differences: review of methods. J Rheumatol.

[16] Amrhein V, Greenland S, McShane B (2019). Scientists rise up against statistical significance. Nature..

[17] Cook JA, Hislop J, Adewuyi TE, Harrild K, Altman DG, Ramsay CR, Fraser C, Buckley B, Fayers P, Harvey I, Briggs AH, Norrie JD, Fergusson D, Ford I, Vale LD (2014). Assessing methods to specify the target difference for a randomised controlled trial: DELTA (Difference ELicitation in TriAls) review. Health Technol Assess.

[18] Cook JA, Julious SA, Sones W, Hampson LV, Hewitt C, Berlin JA, Ashby D, Emsley R, Fergusson DA, Walters SJ, Wilson ECF, MacLennan G, Stallard N, Rothwell JC, Bland M, Brown L, Ramsay CR, Cook A, Armstrong D, Altman D, Vale LD (2018). DELTA^2^ guidance on choosing the target difference and undertaking and reporting the sample size calculation for a randomised controlled trial. BMJ..

[19] Davies HT, Crombie IK, Tavakoli M (1998). When can odds ratios mislead?. BMJ..

[20] Knol MJ, Le Cessie S, Algra A, Vandenbroucke JP, Groenwold RH (2012). Overestimation of risk ratios by odds ratios in trials and cohort studies: alternatives to logistic regression. CMAJ..

[21] Naylor CD, Chen E, Strauss B (1992). Measured enthusiasm: does the method of reporting trial results alter perceptions of therapeutic effectiveness?. Ann Intern Med.

[22] Bath PM, Lees KR, Schellinger PD, Altman H, Bland M, Hogg C, Howard G, Saver JL, European Stroke Organisation Outcomes Working G (2012). Statistical analysis of the primary outcome in acute stroke trials. Stroke..

[23] Copsey B, Thompson JY, Vadher K, Ali U, Dutton SJ, Fitzpatrick R, Lamb SE, Cook JA (2018). Sample size calculations are poorly conducted and reported in many randomized trials of hip and knee osteoarthritis: results of a systematic review. J Clin Epidemiol.

