# Supplementary material for: Current practice in analysing and reporting binary outcome data—a review of randomised controlled trial reports
Source: BMC Med. 2020 Jun 8;18:147. doi: 10.1186/s12916-020-01598-7 (PMC7278160; doi:10.1186/s12916-020-01598-7)
Supplement: Supplementary file 1 — Additional file 1. Summary of search strategy and papers identified. [file 12916_2020_1598_MOESM1_ESM.docx]

**Table S1: Summary of search strategy and papers identified**

| # ▲ | Searches | Results |
| --- | --- | --- |
| 1 | randomized controlled trial.pt. or random*.ti. or (random* and (trial or placebo)).ti,ab. | 653207 |
| 2 | (2019 Jan* or 2019 01* or 201901*).dp,ep. | 147282 |
| 3 | 1 and 2 | 4113 |
| 4 | (systematic review or meta-analysis or review or comment or letter or editorial or news).pt. or (review or meta-analysis or metaanalysis).ti. | 4556115 |
| 5 | exp animals/ not humans.sh. | 4571231 |
| 6 | 4 or 5 | 8906511 |
| 7 | 3 not 6 | 3437 |

Key: ab – abstract; dp – date of publication; ep –electronic date of publication ; pt – publication type; sh – subject heading; ti – title; *- truncation
