# Supplementary material for: Current practice in analysing and reporting binary outcome data—a review of randomised controlled trial reports
Source: BMC Med. 2020 Jun 8;18:147. doi: 10.1186/s12916-020-01598-7 (PMC7278160; doi:10.1186/s12916-020-01598-7)
Supplement: Supplementary file 2 — Additional file 2. List of items extracted. [file 12916_2020_1598_MOESM2_ESM.docx]

**Table S2: List of items extracted**

| **Column title in extraction spreadsheet** | **Comments/ clarification** |
| --- | --- |
| ID |  |
| Person performing extraction |  |
| Author |  |
| Title |  |
| Journal |  |
| DOI |  |
| Data extracted | Yes/no  i.e. no, if article meets one of the exclusion criteria below |
| If not, specify why | - not binary data - survival analysis - >2 arms - feasibility/ pilot study - Full manuscript not available - unclear what primary endpoint is - Secondary analysis - Cluster trial - Cross-over trial   Other |
| Other reason |  |
| Main disease area | Current list includes:   - Alternative medicine - Anaesthesia - Cardiology - Critical Care - Dentistry - Dermatology - Endocrinology - Gastroenterology - Geriatrics - Haematology / Immunology - Infectious diseases - Musculoskeletal - Nephrology - Neurology - Nutrition & weight loss - Obstetrics / Gynaecology - Oncology - Ophthalmology - Otolaryngology (ENT) - Paediatrics - Pharmacology - Physiology - Psychiatry / Psychology - Public health/ policy intervention - Radiology - Respiratory - Rheumatology - Surgery - Urology   Choose depending on authors’ description of the study population. If none fits, leave blank and clarify in “other disease area” |
| Secondary disease area |  |
| Other disease area |  |
| Multicentre trial | Yes/no/ unclear |
| Main funding source | Public/charity/industry/other/no funding stated  Currently, “no funding stated” use used where this information is missing, and where authors declare that no funding has been received.  If it is clear that no funding was received, please also add this to in the column “other funding” |
| Additional key funding | As above |
| Other funding |  |
| Trial design question | - Superiority (there may be a typo in the excel spreadsheet – but let’s keep using it anyway) - non-inferiority - equivalence - other - unclear |
| Outcome type | - Dichotomisation (of continuous variable) - One-off event (such as stroke, death) - Any one of repeated measures (first migraine attack) - Composite endpoint (should be clearly specified as such) - Other |
| Other outcome type |  |
| N randomised to control |  |
| N randomised to intervention |  |
| N total | Excel spreadsheet with calculate this as the total randomised to control and intervention. Overwrite if only total available. |
| Events in control | Leave blank if not clear (I had one case where only the % of participants with events was mentioned – but because it was not clear how many participants had withdrawn/were lost, I could not infer how many events there were) |
| Events in intervention |  |
| Total events | Excel spreadsheet with calculate this as the total number of events in control and intervention. Overwrite if only total available. |
| Missing data: Is it clear how many primary outcome data are missing? | This may not be obvious for all studies. Propose to scan text, numbers included in analysis (unless there are other reasons for exclusion from the analysis)  Just because a participant was lost to follow-up at some point does not mean they no events will have been recorded for them earlier on in the trial. |
| Missing data control |  |
| Missing data intervention |  |
| Total missing data | Excel spreadsheet with calculate this as the total with missing data in control and intervention. Overwrite if only total available. |
| N analysed in control | Number of participants included in principal analysis in each arm |
| N analysed in intervention |  |
| Total N analysed | Excel spreadsheet with calculate this as the total analysed in control and intervention. Overwrite if only total available. |
| Abstract: relative effect reported | Yes/no |
| Abstract: relative Metric | Only applicable if relative effect has been reported   - Risk ratio - Odds ratio - Percentage increase or decrease - Other |
| Abstract: Other relative metric |  |
| Abstract: CI for relative metric reported | Yes/no  (always answer – there may be a CI without an effect size) |
| Abstract: p-value for relative effect reported | Yes/no  Indicate in a p-value was given for the relative effect  (always answer – there may be a p-value without an effect size) |
| Abstract: Absolute effect reported | Yes/no |
| Abstract: absolute effect metric | Only applicable if absolute effect has been reported   - Risk difference - Other |
| Abstract: details for other absolute effect metric |  |
| Abstract: CI for absolute effect reported | Yes/no  (always answer – there may be a CI without an effect size) |
| Abstract: p-value for absolute effect reported | Yes/no  Indicate in a p-value was given for the relative effect  (always answer – there may be a p-value without an effect size) |
| Abstract: p-value reported for chi-squared style test | This was a new addition.  Please indicate if any p-values were from a chi-squared-style test.  (so this could be yes in addition to one of the previous p-value questions, or no if either no p-value was reported, or it did not come from a chi-squared test) |
| Abstract: statistical method for primary results | - Logistic regression - Chi-squared - Poisson - Probit - Tobit - GEE - Binomial regression - Log linear regression - McNemar - Looking at confidence limits - Other - Not reported   Where no details were given in abstract, select “not reported” |
| Abstract: Other statistical method for primary results |  |
| Main text: relative effect reported | Yes/no |
| Main text: relative Metric | Only applicable if relative effect has been reported   - Risk ratio - Odds ratio - Percentage increase or decrease - Other |
| Main text: Other relative metric |  |
| Main text: CI for relative metric reported | Yes/no  (always answer – there may be a CI without an effect size) |
| Main text: p-value for relative effect reported | Yes/no  Indicate in a p-value was given for the relative effect  (always answer – there may be a p-value without an effect size) |
| Main text: Absolute effect reported | Yes/no |
| Main text: absolute effect metric | Only applicable if absolute effect has been reported   - Risk difference - Other |
| Main text: details for other absolute effect metric |  |
| Main text: CI for absolute effect reported | Yes/no  (always answer – there may be a CI without an effect size) |
| Main text: p-value for absolute effect reported | Yes/no  Indicate in a p-value was given for the relative effect  (always answer – there may be a p-value without an effect size) |
| Main text: p-value reported for chi-squared style test | Please indicate if any p-values were from a chi-squared-style test.  (so this could be yes in addition to one of the previous p-value questions, or no if either no p-value was reported, or it did not come from a chi-squared test) |
| Main text: statistical method for primary results | - Complete cases analysis (CCA) - Multiple imputation (MI) - Worst-case analysis - Unclear - Other   Where no details were given, select “unclear” |
| Main text: statistical method for primary results Other methods | - Logistic regression - Chi squared - Poisson - Probit - Tobit - GEE - Binomial regression - Log linear regression - McNemar - Looking at confidence limits - Other - Not reported   Where no details were given, select “not reported” |
| Main text: Adjustment used in principal analysis model | - adjusted - unadjusted - both - unclear   (it should not really be both in a single, principal analysis – but I have seen reports to this effect) |
| Main text: Were further analysis of primary outcome performed | Yes/no |
| Main text: Further analyses include: Subgroups | Yes/no  This includes any analyses were differences between subgroups were considered  Only applicable if “further analyses” were performed |
| Main text: was additional adjusted analysis performed where primary principal was unadjusted, or vice versa? | Yes/no |
| Main text: Further analyses include: change definition of covariates | Yes/no  Were covariate changed, or new ones added, some removed. |
| Main text: Further analyses include: Different classification of endpoint | Yes/no  i.e. consider continuous variable (where main analysis used dichotomisation, different classifications of outcomes, such as considering also less severe outcomes, etc.)) |
| Main text: Further analyses include: Other | Anything else that is considered relevant. |
| Was there any missing outcome data? | Yes/no  Even if we do not know how much data are missing exactly, we should know if at least some primary follow-up data were unavailable. |
| How was missing data handled in principal analysis | - Complete cases analysis (CCA) - Multiple imputation (MI) - Worst-case analysis - Unclear - Other |
| Was appropriate sensitivity analyses for missing data performed? | Yes/ no  The literature defines this as an analysis whereby the underlying assumptions about the missing data mechanism are changed – particularly with a view to missing not at random (MNAR).  For example, a CCA assumes data are missing at random (MAR) for adjusted analyses, or missing completely at random (MCAR) for unadjusted analyses.  Assume the principal analysis was a CCA.  A sensitivity analysis using MI, which also makes a MAR assumption, would be categorised as “no”, while a sensitivity analysis based on a worst-cases analysis would be categorised as “yes”. |
| Any comments | Indicate if there was anything unusual about the study, or any questions you have. |
